# The Disappearing Act: A Case of Emphysematous Pyelonephritis and a Vanishing Kidney Allograft

**DOI:** 10.1155/crit/3760606

**Published:** 2026-02-04

**Authors:** Laura Imarhiagbe, Manish Talwar, Barry M. Wall, Vasanthi Balaraman, Zeina Khairy, Aimen Liaqat

**Affiliations:** ^1^ Department of Medicine, University of Tennessee Health Science Center, Memphis, Tennessee, USA, tennessee.edu; ^2^ Department of Medicine, Veterans Affairs Medical Center, Memphis, Tennessee, USA, va.gov; ^3^ Department of Pathology, Methodist University Hospital, Memphis, Tennessee, USA, methodisthealth.org

## Abstract

Emphysematous pyelonephritis is an acute, severe infection of the kidneys with gas accumulation in the kidneys and adjacent tissues, which is associated with significant morbidity and mortality. Primarily seen in native kidneys, it is relatively rare in renal allograft, despite the immunosuppressed state of transplant recipients and is associated with a high risk of graft loss. Risk factors include urinary tract abnormalities, urological procedures, diabetes mellitus, female gender, and the postmenopausal state. We report a transplant recipient with rapid progression of acute pyelonephritis to emphysematous pyelonephritis and eventually required a transplant nephrectomy. Management is geared towards early detection, judicious antibiotic therapy, repeat imaging, and timely intervention.

## 1. Introduction

Urinary tract infections (UTI) remain a common clinical problem in kidney transplant recipients, with the highest incidence occurring within the first 3–6 months posttransplant and UTIs account for 30% of hospital admissions for sepsis in kidney recipients [1].

Emphysematous pyelonephritis is a severe infection of kidney parenchyma characterized by necrosis and gas accumulation in the kidney parenchyma, adjacent tissues, and urinary collecting system [2–4]. It is a life‐threatening disease with major risk factors including poorly controlled diabetes mellitus, immunosuppression, and urinary tract obstruction [5]. In 70% of cases, *Escherichia coli* is the causative pathogen, with *Klebsiella pneumoniae*, *Candida* species, and *Pseudomonas aeruginosa* occurring less frequently [4]. The risk of UTI in renal transplant patients is higher than in the general population, arising from immunosuppressive therapy, peri‐ and post‐operative instrumentation of the urinary tract, as well as comorbidities such as diabetes mellitus, which frequently coexist in transplant recipients [6]. UTI, notably pyelonephritis, has been found to be a common cause of hospitalization postkidney transplantation [7].

In this report, we present a case of severe emphysematous pyelonephritis with a very rapid evolution to complete kidney allograft destruction, resulting in a sonographically vanishing kidney allograft.

## 2. Case Presentation

A 60‐year‐old African‐American female with a past medical history of end‐stage renal disease secondary to diabetic nephropathy received a deceased donor kidney transplant in June 2023 at the Methodist University Hospital, Memphis. The early postoperative course was complicated by the development of a ureteral stricture with resultant hydronephrosis on postoperative day 2, necessitating surgical revision with ureteroneocystostomy and stent placement. Subsequently, the hydronephrosis completely resolved, allograft function improved with a nadir serum creatinine of approximately 2 mg/dL, and the stent was removed at the sixth week. She had poorly controlled diabetes despite escalation of insulin therapy in the months following the kidney transplantation. This poorly controlled diabetes necessitated her conversion from initial therapy with tacrolimus to cyclosporine to mitigate the tacrolimus‐induced pancreatic islet cell injury, with eventual goal to convert her to belatacept and completely wean off calcineurin inhibitors. She was subsequently maintained on quadruple immunosuppression therapy with cyclosporine (average trough levels of 150 ng/mL), mycophenolic acid 360 mg twice daily, prednisone and monthly belatacept (5 mg/kg) infusions. Posttransplant, she had two additional hospitalizations: one for uncontrolled hyperglycemia and *E. coli* UTI at the third month and one for *Klebsiella* UTI at the fifth month. These UTI episodes resolved with antibiotics. At the 8‐month posttransplant, she presented to the emergency room at the Methodist University Hospital, Memphis, with acute onset of fever, nausea, vomiting, and diarrhea. At presentation, she was febrile, tachycardic, normotensive and had no tenderness over the allograft. Urinalysis was positive for pyuria and leukocyte esterase. She was admitted for severe sepsis secondary to UTI, complicated by diabetic ketoacidosis (DKA) and acute kidney injury, requiring dialysis initiation on day of presentation. Blood and urine cultures were positive for *E. coli*. Computed tomography (CT) of the abdomen and pelvis at admission was unremarkable, demonstrating atrophic native kidneys and a lower quadrant transplanted kidney with normal features (Figure 1a). Her initial management included sepsis protocol with broad spectrum antibiotics and immediate management of her DKA per protocol in the intensive care unit, and all immunosuppressive medications were held. She had rapid deterioration in her clinical state within 12 h of presentation, necessitating pressor support. On the third day of admission an allograft ultrasound was done due to worsening clinical condition and deteriorating allograft function. No sonographically visible kidney allograft could be identified on this ultrasound. A repeat CT abdomen/pelvis revealed findings of significant liquefaction of the transplant kidney replaced by pockets of air and abscess formation (Figure 1b), necessitating a percutaneous perinephric drain placement.

Figure 1(a) Axial computed tomography imaging on Feb 29, 2024, showing normal appearance of the transplant kidney. (b) Repeat axial CT imaging on March 2, 2024, depicting pockets of air and abscess formation.

**Figure:**
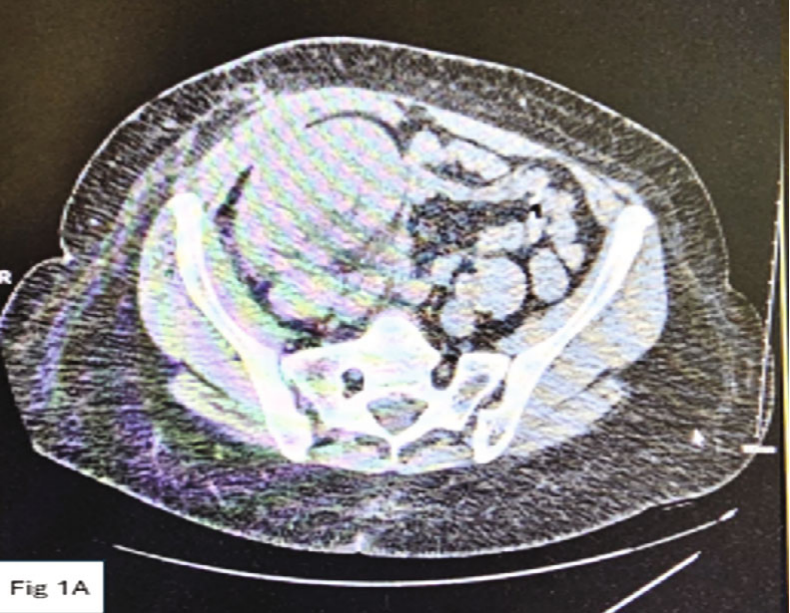
(a)

**Figure:**
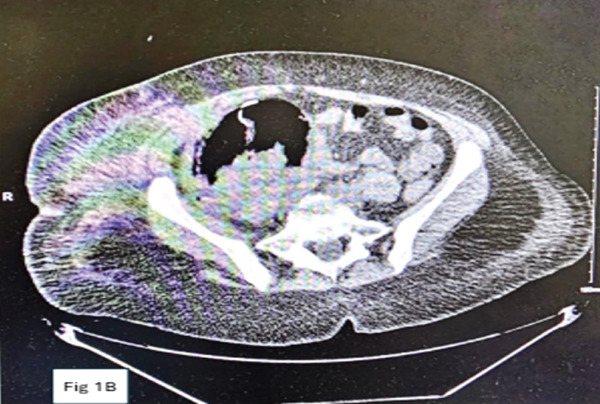
(b)

**Figure 2:**
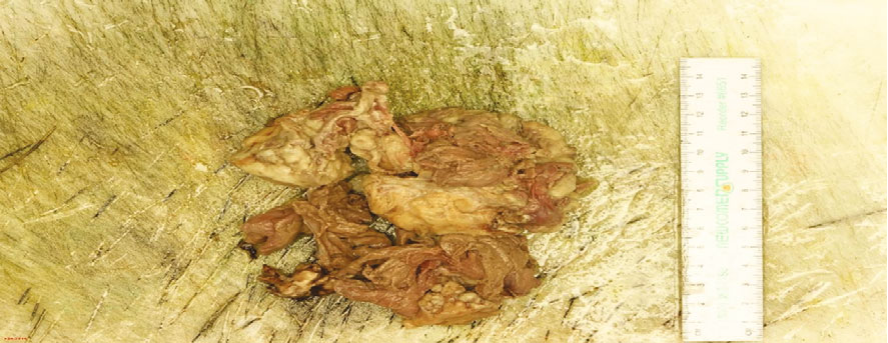
Explant of kidney allograft showing fragmentation with liquified necrosis of the lower pole and solid component of the upper pole.

She failed to improve clinically and radiologically, with repeat CT abdomen/pelvis 4 days later showing extensive emphysematous changes within the transplant kidney. She underwent a transplant nephrectomy, with intraoperative findings of a fragmented transplant kidney with liquified necrosis of the lower pole and solid component of the upper pole (Figure 2). Surgical cultures of the explanted kidney tested positive for vancomycin‐resistant *Enterococcus faecium*.

**Figure 3:**
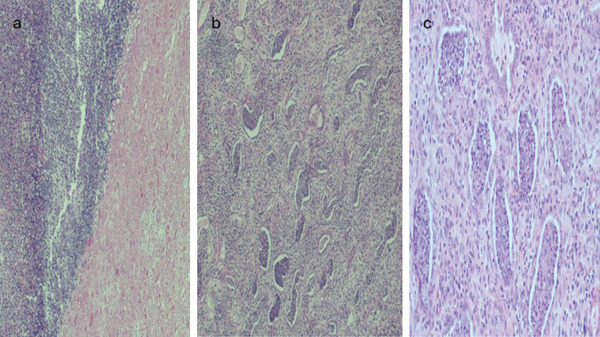
(a) Cortical abscess (blue arrows) with marked neutrophilic inflammation and necrosis (black arrows) [H&E 2x]. (b) Patchy suppurative inflammation with tubular lumina and tubular microabscesses and interstitial acute and chronic inflammation [H&E 4x]. (c) High power view of tubular microabscesses (blue arrows) [H&E 10x].

Histology showed extensive abscesses, patchy renal cortical necrosis, and mild to moderate arteriosclerosis (Figures 3a, 3b, and 3c). Eventually, targeted antibiotic therapy with ceftriaxone and linezolid resulted in complete resolution of her infection. She was discharged in stable condition on regular hemodialysis and relisted for kidney transplant.

## 3. Discussion

Our patient presented with sepsis and bacteremia related to acute pyelonephritis in the allograft. Initial assessments showed no evidence of urinary tract obstruction or emphysematous pyelonephritis at the time of admission. Following rapid clinical deterioration and decline in allograft function, repeat imaging studies done demonstrated a “vanishing kidney” on ultrasonography and extensive emphysematous pyelonephritis on CT imaging. The uniqueness of this case was the rapidity of progression of pyelonephritis (Table 1) with normal sonographic features at presentation and severe liquefactive necrosis of her allograft on repeat imaging within 48 h of presentation.

**Table 1:** Timeline of events.

**02/29/2024**	**ED with fever, vomiting, and diarrhea. CT abdomen/pelvis unremarkable allograft kidney**
03/02/2024	Allograft USS with no sonographically visible kidney
	CT abdomen/pelvis with liquefaction of the transplant kidney replaced by pockets of air and abscess formation
03/03/2024	Percutaneous drain placed into perirenal space of transplant kidney
03/07/2024	CT abdomen/pelvis with extensive emphysematous changes within the transplant kidney
03/10/2024	Transplant nephrectomy

Vanishing kidney is an advanced presentation of emphysematous pyelonephritis where a kidney previously visible sonographically disappears as layers of encasing gas pockets block penetration of ultrasonographic waves [8]. A conservative management approach, with intravenous antibiotics and percutaneous drainage of the abscess, was initially used for our patient with the goal of preserving the renal allograft. However, her clinical deterioration and imaging findings of extensive liquefaction of the renal allograft necessitated an emergent allograft nephrectomy and return to maintenance hemodialysis.

Although UTI are common in kidney transplant recipients, emphysematous pyelonephritis, primarily seen in native kidneys, is relatively rare in renal allograft, even though transplant recipients are more susceptible due to their immunosuppressed state. The risk factors for postkidney transplant UTI are multifactorial, including older age, diabetes, female gender, ureteric stenosis, ureteral stents as well as other instrumentation [1]. In our patient, besides her immunosuppressed state, she had other risk factors of early ureteric stenosis with hydronephrosis requiring ureteroneocystostomy and stent placement, poorly controlled diabetes, and her postmenopausal state.

Al‐Geizawi et al. [4], described a transplant patient who likely presented in Stage 3 of the Huang and Tseng staging for native emphysematous pyelonephritis [2], who improved with intensive medical management. Similar to our patient, cases reported by Ambinder et al. [3] and Abu Jawdeh et al. [5] required transplant nephrectomy for deteriorating clinical course. In renal transplant patients, emphysematous pyelonephritis is associated with high risk of graft loss and return to maintenance dialysis, as well as higher mortality rates [ 3, 9].

A high index of suspicion upon nonvisualization of kidney allograft on ultrasound warrants immediate confirmation with CT imaging, as early diagnosis and timely intervention are crucial for potential graft salvage and for recognition of this life‐threatening complication of pyelonephritis.

Classification for emphysematous pyelonephritis (Table 2) is based on CT findings and includes the well‐known staging system for native kidneys by Huang and Tseng [2], as well as a proposed classification by Al‐Geizawi et al. [4], for renal allografts which take into account the absence of an investing Gerotas fascia around the transplanted kidney [4]. Our patient met the criteria as Huang Class 4 or Al‐Geizawi Stage 3.

**Table 2:** Huang and Tseng classification of native emphysematous pyelonephritis and Al‐Geizawi proposed staging system for emphysematous pyelonephritis in renal allografts.

**Huang native kidney classification**	**Al-Geizawi renal allograft staging**
Class 1: Gas confined to the collecting system only.	Stage 1: Gas in the collecting system.
Class 2: Gas confined in the renal parenchyma	Stage 2: Gas replacing < 50% of renal parenchyma, with minimum spread to the surrounding tissues. Sepsis rapidly controlled.
Class 3a: Gas or abscess extends to the perinephric space.	Stage 3: Gas replacing >50% of renal parenchyma or extensive spread of infection in the perinephric area; or patient with evidence of multiple organ failure, uncontrolled sepsis, or shock.
Class 3b: Gas or abscess extends beyond the Gerota fascia into the pararenal space.	
Class 4: Bilateral renal involvement or involvement of a solitary kidney	

Therapeutic approaches for management of emphysematous pyelonephritis have been a subject of controversy, with opinions split between renal conservative management utilizing intravenous antibiotics and percutaneous drainage for earlier stages or nephrectomy, which was previously considered the gold standard [10, 11]. Recently, with early diagnosis and improvement in management techniques, there has been a shift towards kidney conserving management utilizing antibiotics, percutaneous drainage and stenting [4, 12–14]. Nephrectomy is now reserved for more severe cases [3] or failure of conservative management, as was the case with our patient.

The American Society of Transplantation Infectious Diseases Community of Practice guidelines [1] for management of UTI in kidney transplant patients is for screening and treatment of asymptomatic bacteriuria in the first 2 months posttransplant, and early judicious use of antibiotics for symptomatic UTIs, taking into consideration patients′ previous history of organisms, and in complicated and worsening infection, utilization of repeat imaging of the genitourinary tract to exclude progression to renal/perinephric abscess, emphysematous pyelonephritis or obstruction. These complications usually require multidisciplinary management and interventional procedures for drainage or relief of obstruction if present.

With UTIs being a common clinical problem in kidney transplant recipients, prevention of recurrent UTIs (defined as ≥ 2 UTIs in a 6‐month period or ≥ 3 UTIs in a 12‐month period) becomes paramount with recurrent UTIs. Present management guidelines do not recommend treatment for asymptomatic bacteriuria beyond the first 2 months postkidney transplant. Our patient had a total of three UTIs in 8‐month posttransplant. Guidelines recommend that kidney transplant recipients with recurrent UTIs will benefit from interventions targeting the risk factors as well as lifestyle modifications. These include correction of structural and functional anomalies of the urinary tract, infection prevention measures including proper perineal hygiene and use of topical vaginal estrogens in postmenopausal female recipients [1].

## 4. Conclusion

Emphysematous pyelonephritis is an aggressive form of pyelonephritis, though relatively uncommon in immunocompromised renal transplant recipients, with significant morbidity and mortality and a high risk of allograft loss and return to dialysis. It requires a high index of suspicion when transplant recipients present with worsening renal allograft failure in the setting of acute pyelonephritis as it can rapidly transform to an emphysematous pyelonephritis, for timely and judicious use of broad‐spectrum antibiotics. Diagnosis requires imaging, often multiple repeats, with clinical deterioration despite appropriate antibiotic therapy. Management requires a multidisciplinary approach involving urologists, interventionists, nephrologists, transplant surgeons, and infectious disease specialists.

## Consent

Informed consent was taken from the patient for submission of this report.

## Conflicts of Interest

The authors declare no conflicts of interest.

## Author Contributions

Laura Imarhiagbe: conceptualization, data collection, and writing original draft. Aimen Liaqat: conceptualization, supervision and review and editing (equal). Barry M. Wall: critical review and editing (equal). Manish Talwar: review and editing (equal) and data acquisition. Vashanti Balaraman: review and editing (equal). Zeina Khairy: formal analysis and review and editing of histology.

## Funding

No funding was received for this manuscript.

## Data Availability

The data supporting the findings of this case report are not publicly available due to patient confidentiality considerations. Deidentified data may be made available from the corresponding author upon reasonable request and with appropriate institutional and ethical approvals.
